# The clinicopathological characteristics, prognosis and immune microenvironment mapping in MSI-H/MMR-D endometrial carcinomas

**DOI:** 10.1007/s12672-022-00466-5

**Published:** 2022-03-03

**Authors:** Yu-e Guo, Yin Liu, Wei Zhang, Heng Luo, Ping Shu, Guofang Chen, Yuping Li

**Affiliations:** 1 Department of Pharmacy, Shanghai Pulmonary Hospital, Tongji University School of Medicine, 507 Zhengmin Road, Shanghai, 200433 China; 2 Department of Laboratory Medicine, Shanghai Pulmonary Hospital, Tongji University School of Medicine, Shanghai, China; 3 Clinical and Translational Research Center, Shanghai First Maternity and Infant Hospital, Tongji University School of Medicine, 2699 West Gaoke Road, Shanghai, 201204 China

**Keywords:** Microsatellite instability (MSI), Mismatch repair (MMR), Endometrial carcinoma, Immune infiltration, Single-cell RNA-seq analysis

## Abstract

Endometrial cancer had a relatively high prevalence of MMR deficiency. MMR-D/MSI-H endometrial cancer patients are suggested to be potential beneficiaries of PD-1/PD-L1 inhibitor therapy. Here, we explored the prognostic value of MSI subtype in endometrial cancer and its correlation with immune environment. Based on expression and clinical data of 78 POLE, 123 MSI and 299 Other EC samples from the TCGA-UCEC project, we found that the MSI tumors were identified more often in early stage, had a lower age, better patient survival, enriched CD8^+^ T cells, and regulatory T cells and less M2 macrophages and activated dendritic cells than the Other group, and shared a relatively similar expression profile with POLE group by differential analysis. In addition, we established the immune landscape of an MMR-D endometrial cancer tissue using unbiased single-cell RNA-seq analysis of 3371 cells. By immunohistochemistry analysis, we found that the MMR-D tumors showed a higher trend of CD20^+^ B cells infiltration. Our study might expand our understanding of the role of immune subsets in MSI endometrial carcinomas and provide guidance of immunotherapy for endometrial cancer.

**Supplementary Information:**

The online version contains supplementary material available at 10.1007/s12672-022-00466-5.

## Introduction

Endometrial carcinomas (ECs) have been broadly categorized into two subtypes according to clinicopathologic characteristics, hormone receptor expression and grade in the past 30 years [1]. Type I tumors account for 70% to 80% of ECs, and are endometrioid, hormone-receptor-positive, low-grade ECs with a good prognosis. Type II ECs are non-endometrioid, hormone-receptor-negative, high-grade tumors, which exhibit higher risk of metastasis and poor outcomes [1, 2]. However, the prognostic value of this dualistic classification remains limited, proper subtyping is critical for selecting appropriate treatment [1, 3]. In 2013, molecular classification by The Cancer Genome Atlas characterized endometrioid and serous endometrial cancer into four distinct molecular subgroups: POLE ultramutated, microsatellite instability hypermutated (MSI-H), copy number low (endometrioid) and copy number high (serous-like) [4]. Molecular classification is one of the most important developments in the study of endometrial carcinoma in recent years. The ESGO/ESTRO/ESP (the European Society of Gynaecological Oncology (ESGO), the European Society for Radiotherapy & Oncology (ESTRO) and the European Society of Pathology (ESP)) 2020 classification of endometrial carcinoma revised the pathological subtypes and integrated the molecular typing of endometrial cancer [5].

MSI-H endometrial cancer patients are potential beneficiaries of PD-1/PD-L1 inhibitor therapy [6]. MSI is usually as a result of defects in the mismatch repair (MMR) system, a group of enzymes that is responsible for monitoring and repairing the error incorporations in microsatellites [7]. The MMR genes consist of MLH1, MSH2, MSH6 and PMS2. Lynch syndrome results from germline mutations in four MMR genes [8]. Determination of the MSI phenotype in endometrial carcinoma patients by testing MMR status/microsatellite instability (MSI) has been shown to be relevant for four clinical applications: as a surrogate marker for histopathological typing; for Lynch syndrome screening; helping to predict the EC patient’s prognosis and determine treatment decisions such as the potential utility of immune checkpoint inhibitor therapy. The International Society of Gynecological Pathology (ISGyP) has suggested universal testing for MMR status/MSI routinely in all endometrial carcinoma cases [9].

There are two assays used to determine MMR-deficient/MSI-H phenotype (simply MSI hereafter) in current clinical testing: MMR—immunohistochemistry (IHC), and PCR-based DNA microsatellite instability analysis (MSI-test) [9, 10]. MMR-IHC is now the first-line approach to identify patients with MMR deficiency. MMR-IHC is a widely available, cost-effective and reliable method to assess MMR status, which can provide direct information on the absence of expression of the altered gene/protein. Alternative MSI-test and subsequent reflex *MLH1* hypermethylation, panel testing for germline mutations in > 20 cancer-causing genes (which include the MMR genes) would be undertaken when indicated.

Identification of the MSI phenotype in colorectal cancer has been well used to help determine treatment decisions for targeted immunotherapy. Some studies also have proven that mismatch-repair deficiency can predict PD-1 blockade response in solid tumors [6, 11–13]. Importantly, the UCCN (National Comprehensive Cancer Network) and ESGO/ESTRO/ESP guidelines in 2020 recommended anti-PD-1 targeted therapy for some advanced MSI EC patients [5]. But its clinical effect remains to be well investigated and improved. Thus, owing to the high prevalence of MMR deficiency in endometrial cancer and potential applications of immunotherapy in EC [1, 14], deeper understanding of MMR-D/MSI-H subtype of endometrial cancer is extremely important.

In the present study, we aimed to explore the relationships between the MSI status and EC clinical features, prognosis, mutation profile, immune infiltrates based on The Cancer Genome Atlas (TCGA) data, and to explore the cell landscape of an MMR-D/MSI-H cancer tissue by single cell-RNA analysis.

## Materials and methods

### TCGA data collection and analysis

Gene expression data, somatic mutation profiles, clinical and survival information were downloaded from the TCGA database UCEC project by UCSC Xena [15] (https://xenabrowser.net/datapages/?cohort=GDC%20TCGA%20Endometrioid%20Cancer%20(UCEC)&removeHub=https%3A%2F%2Fxena.treehouse.gi.ucsc.edu%3A443). RNA expression data were normalized and aligned using R software (version 3.6.2; https://www.r-project.org). Patients whose molecular subtype classification information were unknown were excluded from our study. Thus, data of 500 endometrial cancer patients were analyzed in this study.

### Clinicopathological characteristics and prognostic analyses

We explored the association between molecular subtype and age at diagnosed, BMI by Kruskal–Wallis test. The association between molecular subtype and other clinical variables (clinical stage, histologic grade, diabetes, hypertension, menopause state) were explored by chi-square test. Prognostic analysis of clinical variables was performed for overall survival using Kaplan–Meier curves with log-rank test. For all statistical tests, the P values < 0.05 were considered statistically significant.

### Acquisition and analysis of mutation spectra

The Mutation Annotation Format (MAF) data that were generated by using MuTect2 (4.1.1.0) [16] from whole exome sequencing data were downloaded. The R software (version 3.6.2) “maftools” package was used to provide visual mutation spectra. The Oncoplot function was used to generate waterfall plot of the top 50 mutated genes using COSMIC database.

### Differentially expressed genes in three subtype groups and enrichment analysis

The differential analysis was performed by R software (version 3.6.2) “limma” package (version 3.50.0) [17] with one-way analysis of variance (ANOVA). Bonferroni adjusted P value < 0.05 was used to filter differentially expressed genes (DEGs). The significant DEGs were visualized using R software “pheatmap” package (version 1.0.12). GO (Gene Ontology) and KEGG (Kyoto Encyclopedia of Genes and Genomes) analysis were performed using R package clusterProfiler (version 4.2.1) [18]. P value < 0.05 was considered significantly enriched.

### Estimation of immune composition

The immune scores were obtained using the ESTIMATE algorithm implemented in R packages estimate (version 1.0.13) [19] based on gene expression data. We compared immune scores across the different molecular subtype groups by Kruskal–Wallis test. We estimated the composition profile of 22 immune cell types of 500 samples using the “Cell type Identification by Estimating Relative Subsets Of RNA Transcripts (CIBERSORT)” algorithm (http://cibersort.stanford.edu/) which included the LM22 signature (PMID: 25822800). Different immune cell infiltration level across the different molecular subtype groups were compared by Kruskal–Wallis test. Overall survival analysis was performed using Kaplan–Meier curves with log-rank test for independent immune cells. For all statistical tests, the P values < 0.05 were considered statistically significant.

### Single-cell suspension reparation and 10 × library sequencing

Following resection, a representative tumor fragment was isolated and transferred rapidly to the laboratory for study as previously described [20]. Briefly, tissue was initially cut into segments and subjected to digestion by collagenase type I/II (Thermo Fisher Scientific, USA) and DNAse I (Sigma, USA). The digested pieces were triturated with a 1 ml syringe plunger and passed through a 70 μm cell strainer (Coring, USA). After resuspending in red blood cell lysis buffer (Solarbio, China), live cells were enriched using a Dead Cell Removal kit (Miltenyi Biotec, Germany) as per manufacturer’s instructions. Enriched live cells were washed and counted using a hemocytometer with trypan blue. Cells were then resuspended in PBS containing 0.04% BSA at a concentration of 1 × 10^6^ cells/ml with a viability of > 80% as determined with the Countess. Overall, the entire dissociation procedure took about 2 h from obtaining sample to generating single-cell suspension. The single-cell suspension was then run on the Chromium 10X device (10 × Genomics, USA).

Single-cell library preparation was carried out using Chromium Single cell 3’ Reagent v2 Kits (10 × Genomics, USA) according to the manufacturer’s protocol. Then the library was sequenced on the HiSeq X Ten instruments (Illumina, USA) and 150 bp paired-end reads were generated.

### Single-cell transcriptome data preprocessing and analysis

We used the Cell Ranger software pipeline (version 2.2.0, 10xGenomics) to process raw sequencing data and Seurat (version 2.3.4) [21] R package for downstream analysis as previously described [20]. Briefly, principle component analysis (PCA) was performed for dimensional reduction. Clusters were identified using the Seurat “FindClusters” algorithm. Graph-based clustering results on 20 principle components were visualized in 2-dimension using t-SNE. Cell clusters were annotated to known biological cell types using canonical marker genes. A cluster-specific biomarker was found by “FindAllMarkers” function identified when it was expressed in a minimum of 25% of cells and at a minimum log fold change threshold of 0.25.

### Immunohistochemistry

Tissue sections were collected and fixed in 10% formalin. 5 μM slides were used for immunohistochemistry analysis. The “UltraVision Quanto Detection System HRP DAB” IHC kit (TL-125-QDH, Thermo Fisher Scientific) was used for the tyramide signal amplification according to the manufacturer’s protocol. Primary antibodies used in this assay are as follows: anti-CD20 (M0755, Dako), anti-MLH1 (MAB-0789, MXB Biotechnologies), anti-MSH2 (MAB-0836, MXB Biotechnologies), anti-PMS2 (GT215902, Gene Tech) and anti-MSH6 (MAB-0831, MXB Biotechnologies). Images were taken and quantitative image analysis was performed using ImagePro software. For comparison for CD20 between two groups, statistical evaluation was done by two-tailed Student’s t-test, error bars show standard error of the mean (SEM).

### Statistical analyses

Statistical analyses were performed using GraphPad Prism 5 software and statistical significance was determined by p < 0.05. For TCGA mutation and RNA-seq data, all statistical analysis was performed with R (version 3.6.2).

## Results

### Association between EC molecular subtypes and survival and clinical features

**Fig. 1:**
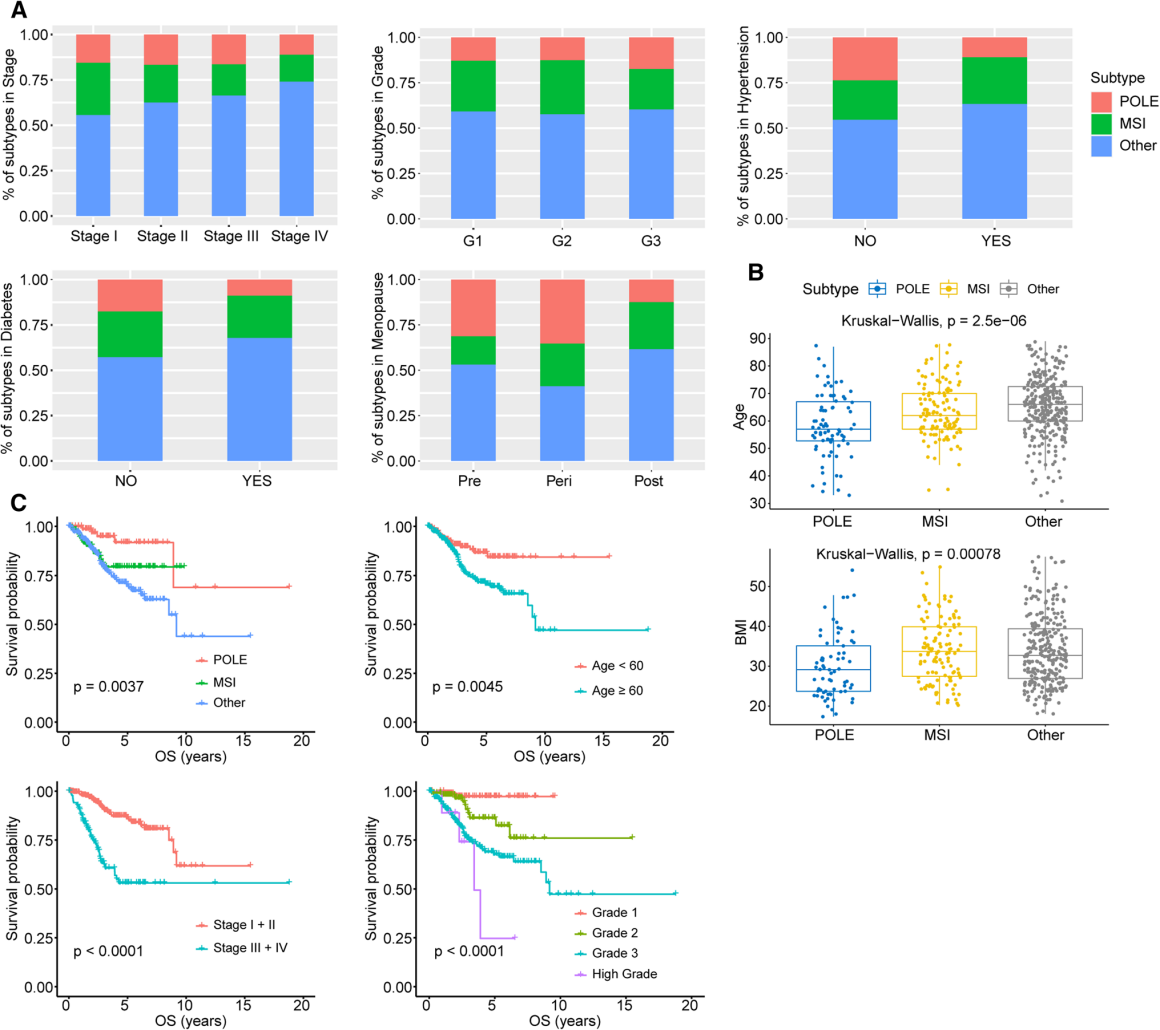
Association between molecular subtypes and clinical variables and survival outcome. **A** The fractions of molecular subtypes in clinical stage, histologic grade, diabetes, hypertension and menopause state. Chi-square test, P values < 0.05 were considered statistically significant. **B** Distribution of age and BMI of EC molecular subtypes. Kruskal–Wallis test, P values < 0.05 were considered statistically significant. **C** Overall survival curve and log-rank test for endometrial cancer patients based on molecular subtype, clinical stage, histologic grade and age classification. P values < 0.05 were considered statistically significant

To explore the relationship between the distinct molecular subtypes of endometrial cancer and the clinical features, we analyzed clinical data of 500 EC patients from TCGA database, including clinical stage, histologic grade, age, history of radiation therapy, diabetes, hypertension, menopause state and BMI (Table 1). The 500 patients were classified into three molecular subgroups: the POLE, MSI and Other subgroup. We discovered that the POLE subgroup has a lower age, BMI, lower fraction of diabetes and hypertension (Fig. 1A, B). The tumors in MSI subgroup were identified more often in the early stage, and had a lower age than the Other subgroup (Fig. 1A, B). These results were consistent with the prognosis analysis between patient survival and molecular subtype, age and clinical stage (Fig. 1A–C). But there is no difference in the proportion of histologic grade across the subtypes (Fig. 1A). The POLE and MSI subtypes, lower age, grade and clinical stage were all correlated with better patient survival (Fig. 1C).

**Table 1:** Clinical characteristics of 500 patients with endometrial cancer from TCGA database

	POLE (N = 78)	MSI (N = 123)	Other (N = 299)	Total (N = 500)
Stage				
Stage I	48 (61.5%)	89 (72.4%)	172 (57.5%)	309 (61.8%)
Stage II	8 (10.3%)	10 (8.1%)	30 (10.0%)	48 (9.6%)
Stage III	19 (24.4%)	20 (16.3%)	77 (25.8%)	116 (23.2%)
Stage IV	3 (3.8%)	4 (3.3%)	20 (6.7%)	27 (5.4%)
Grade				
G1	12 (15.4%)	26 (21.1%)	55 (18.4%)	93 (18.6%)
G2	14 (17.9%)	33 (26.8%)	64 (21.4%)	111 (22.2%)
G3	50 (64.1%)	64 (52.0%)	173 (57.9%)	287 (57.4%)
High grade	2 (2.6%)	0 (0%)	7 (2.3%)	9 (1.8%)
Age				
< 60	45 (57.7%)	50 (40.7%)	72 (24.1%)	167 (33.4%)
≥ 60	31 (39.7%)	73 (59.3%)	227 (75.9%)	331 (66.2%)
Missing	2 (2.6%)	0 (0%)	0 (0%)	2 (0.4%)
Radiation therapy				
No	38 (48.7%)	58 (47.2%)	164 (54.8%)	260 (52.0%)
Yes	38 (48.7%)	54 (43.9%)	118 (39.5%)	210 (42.0%)
Unknown	2 (2.6%)	11 (8.9%)	17 (5.7%)	30 (6.0%)
Diabetes				
No	44 (56.4%)	63 (51.2%)	143 (47.8%)	250 (50.0%)
Yes	8 (10.3%)	21 (17.1%)	61 (20.4%)	90 (18.0%)
Unknown	26 (33.3%)	39 (31.7%)	95 (31.8%)	160 (32.0%)
Hypertension				
No	36 (46.2%)	33 (26.8%)	83 (27.8%)	152 (30.4%)
Yes	23 (29.5%)	54 (43.9%)	133 (44.5%)	210 (42.0%)
Unknown	19 (24.4%)	36 (29.3%)	83 (27.8%)	138 (27.6%)
Menopause				
Pre	10 (12.8%)	5 (4.1%)	17 (5.7%)	32 (6.4%)
Peri	6 (7.7%)	4 (3.3%)	7 (2.3%)	17 (3.4%)
Post	51 (65.4%)	106 (86.2%)	252 (84.3%)	409 (81.8%)
Indeterminate	2 (2.6%)	5 (4.1%)	8 (2.7%)	15 (3.0%)
Unknown	9 (11.5%)	3 (2.4%)	15 (5.0%)	27 (5.4%)
BMI				
< 25	24 (30.8%)	17 (13.8%)	43 (14.4%)	84 (16.8%)
≥ 25	47 (60.3%)	102 (82.9%)	238 (79.6%)	387 (77.4%)
Missing	7 (9.0%)	4 (3.3%)	18 (6.0%)	29 (5.8%)

### Mutation spectra in POLE, MSI, and Other ECs

**Fig. 2:**
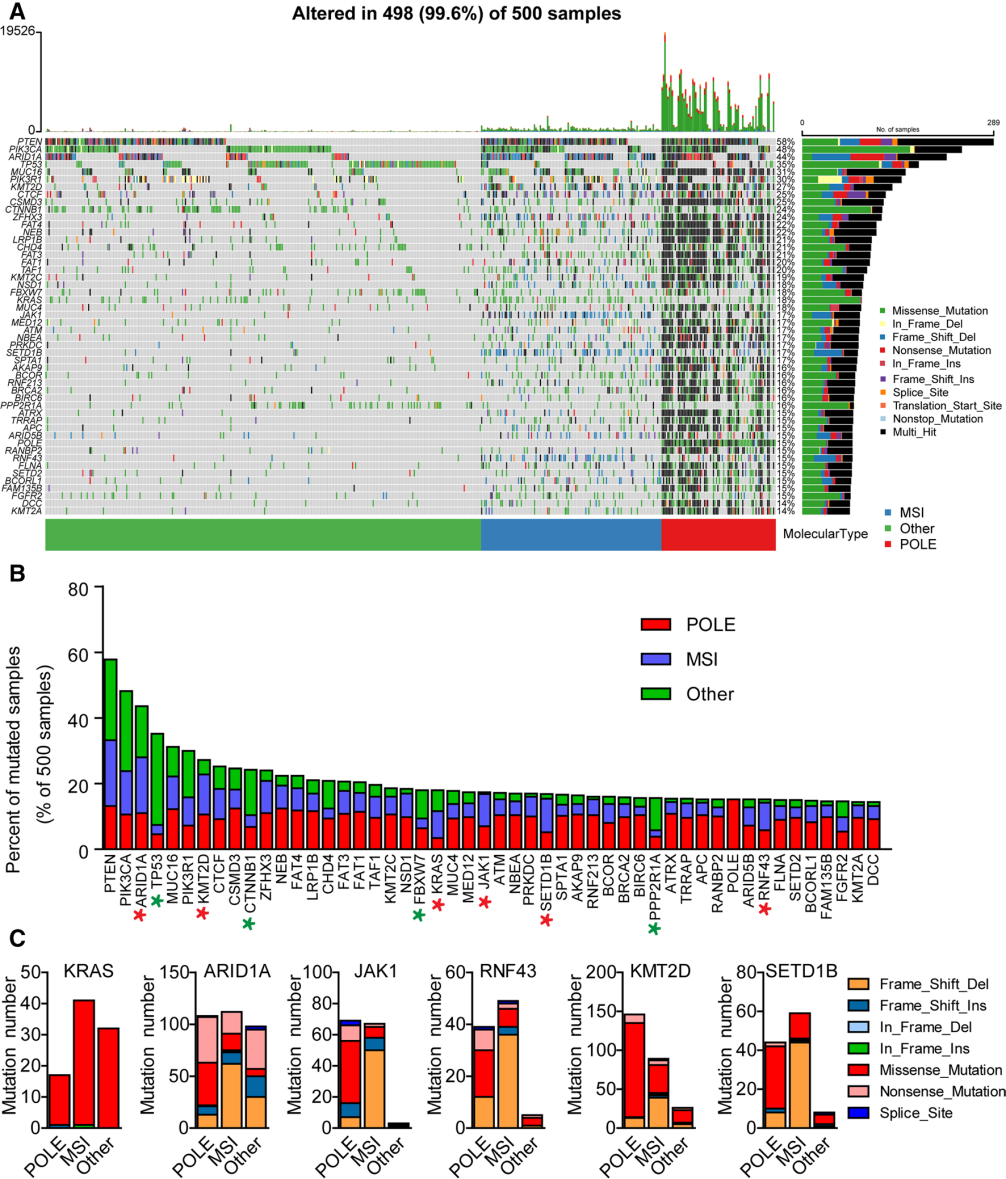
Landscape of somatic mutation profiles in 500 endometrial cancer samples from TCGA database. **A**The waterfall plot shows top 50 mutated genes in each sample. The upper barplot shows the number of mutations in each patient, while the right barplot indicates the number of mutated samples in each gene. **B** Proportion of tumors in three molecular subtypes of each gene. Green stars showed 4 genes with few mutations in MSI group, red stars showed 6 genes with frequent mutations in MSI group. **C** Frequently mutated genes in the MSI subgroup. Shown are the mutation numbers of different variant types in six genes

Tumors with POLE mutation or MSI have been suggested to be hyper or highly mutated. Mutation spectra across 500 EC patients in the TCGA database were analyzed. Most of the genes had higher mutation frequency in the POLE group (Fig. 2A). The MSI group had few mutations in TP53, FBXW7, CTNNB1 and PPP2R1A, which was consistent with previously described (Fig. 2B) [4]. 6 genes showed more frequent mutations in MSI group, including 4 genes (KRAS, ARID1A, JAK1 and RNF43) [4, 22] that have been previously reported in endometrial cancer and 2 novel genes (KMT2D and SETD1B) (Fig. 2B). Except for KRAS with predominantly missense mutation in all groups, other 5 genes showed more frequent frameshift deletions in MSI group than POLE and other groups (Fig. 2C). JAK1 and RNF43 with polymerase slippage-associated deletions have been reported previously [22]. KMT2D and SETD1B are chromatin remodeling-related genes, and appear to help predict the degree of myometrial invasion [23].

### Enriched immune infiltrates in POLE and MSI ECs than Other ECs

**Fig. 3:**
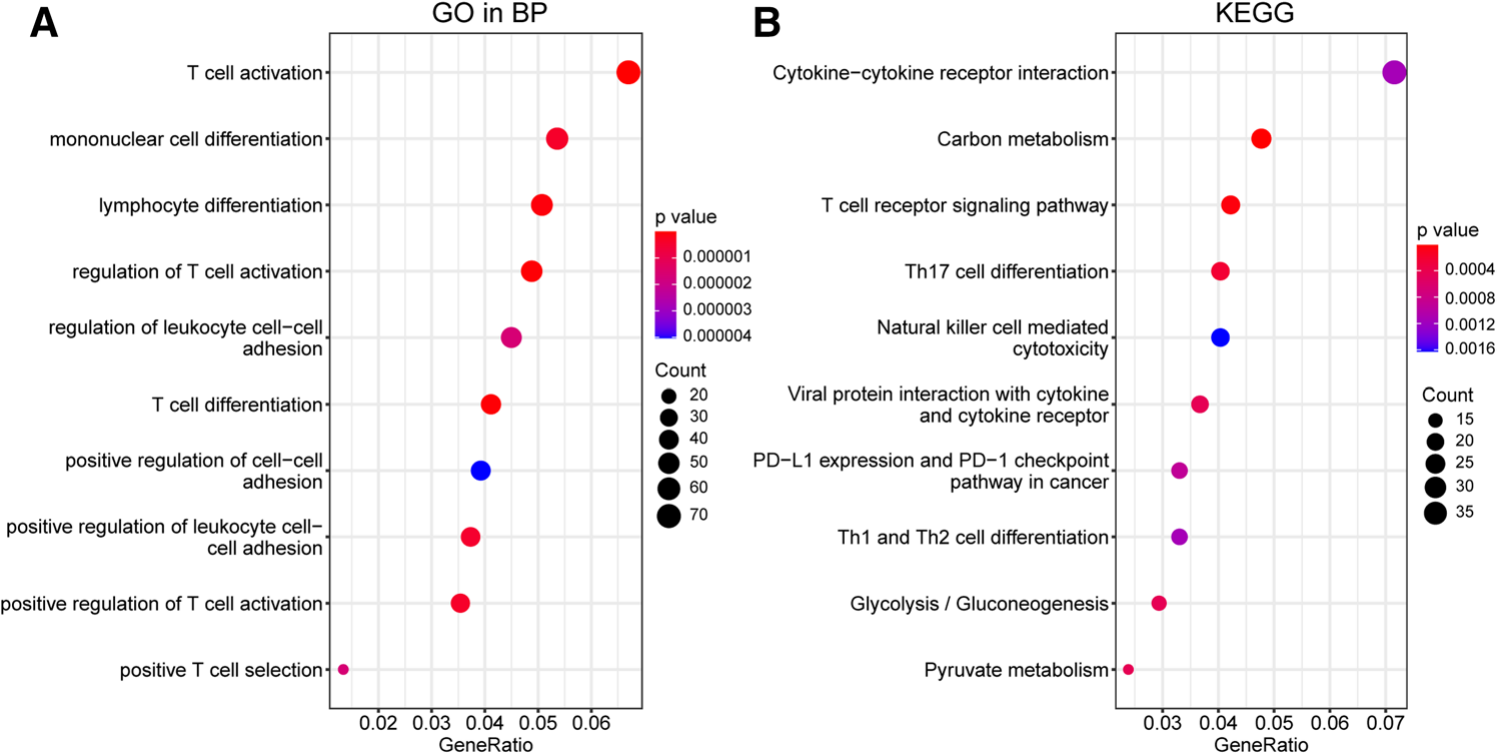
Enrichment analysis of up-regulated genes (DEGs) in POLE and MSI groups compared with Other ECs. **A**, **B** The top 10 of biological processes GO terms (**A**) and KEGG (Kyoto Encyclopedia of Genes and Genomes) pathway (**B**) enriched by 1138 genes those were upregulated in POLE and MSI groups compared with Other ECs. P value < 0.05 was considered significantly enriched

We analyzed the global gene expression profile of 500 EC patients, and identified the differentially expressed genes (DEGs) between the three molecular groups. Differential expression analysis showed that 1138 genes were significantly upregulated in MSI and POLE groups compared with other group (Supplementary table 1, Figure S1). The gene set enrichment analyses (GSEA) using Gene Ontology (GO) terms and Kyoto Encyclopedia of Genes and Genomes (KEGG) terms showed that genes up-regulated in POLE and MSI samples were mainly enriched for immune-related functions, such as T cell activation, T cell differentiation, cytokine-cytokine receptor interaction and T cell receptor signaling pathway (Fig. 3).

**Fig. 4:**
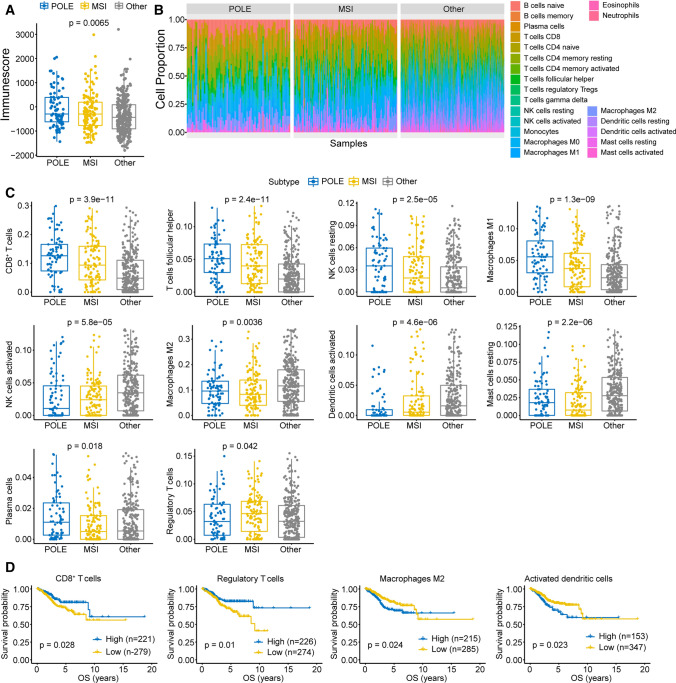
Immune infiltrates in EC samples from TCGA database. **A** Distribution of immune scores of EC molecular type, including POLE, MSI, and Other type. Kruskal–Wallis test. The P values < 0.05 were considered statistically significant. **B** The fractions of 22 immune cell types in 500 EC samples using CIBERSORT algorithm. **C** Relationship between different immune cell types and EC molecular type. Distribution of CD8^+^ T cells, follicular helper T cells, resting NK cells, M1 macrophages, activated NK cells, M2 macrophages, activated dendritic cells, mast cells, plasma cells and regulatory T cells across EC molecular type. Kruskal–Wallis test. The P values < 0.05 were considered statistically significant. **D** Kaplan–Meier curves with log-rank test for two immune cell types associated with good prognosis and two types associated with poor prognosis. The P values < 0.05 were considered statistically significant

POLE mutation or MSI subtypes in cancers indicate hypermutations, and they are suggested to harbor more tumor-specific neoantigens and higher lymphocytes infiltrates [24]. Thus, we calculated the immune scores of all 500 samples using the ESTIMATE algorithm, and found that immune scores of both POLE and MSI EC groups were significantly higher than the Other group (Fig. 4A). To determine the correlation between molecular subtypes and immune composition, we then estimated the composition profile of 22 immune cell types of 500 samples using CIBERSORT algorithm (Fig. 4B). Furthermore, the Kaplan–Meier curve with log-rank test was used to analyze the correlation between each immune infiltrate level and the overall survival (OS) time of EC patients. The fractions of 10 immune cell types varied across molecular subtypes (Fig. 4C). Importantly, both POLE and MSI groups were found to have more CD8^+^ T cells, follicular helper T cells, resting NK cells, M1 macrophages, while less activated NK cells, M2 macrophages, activated dendritic cells and mast cells (Fig. 4C). POLE groups had more plasma cells (Fig. 4C). MSI group had more regulatory T cells (Fig. 4C). Among these immune cell types, high infiltrates level of CD8^+^ T cells, and regulatory T cells were found to be significantly positively related with OS, while high infiltrates level of M2 macrophages and activated dendritic cells were negatively related with OS (Fig. 4D). These results were consistent with better clinical outcomes in POLE and MSI EC groups than Other group.

### Cell mapping of immune ecosystem in an MMR-D EC sample

**Fig. 5:**
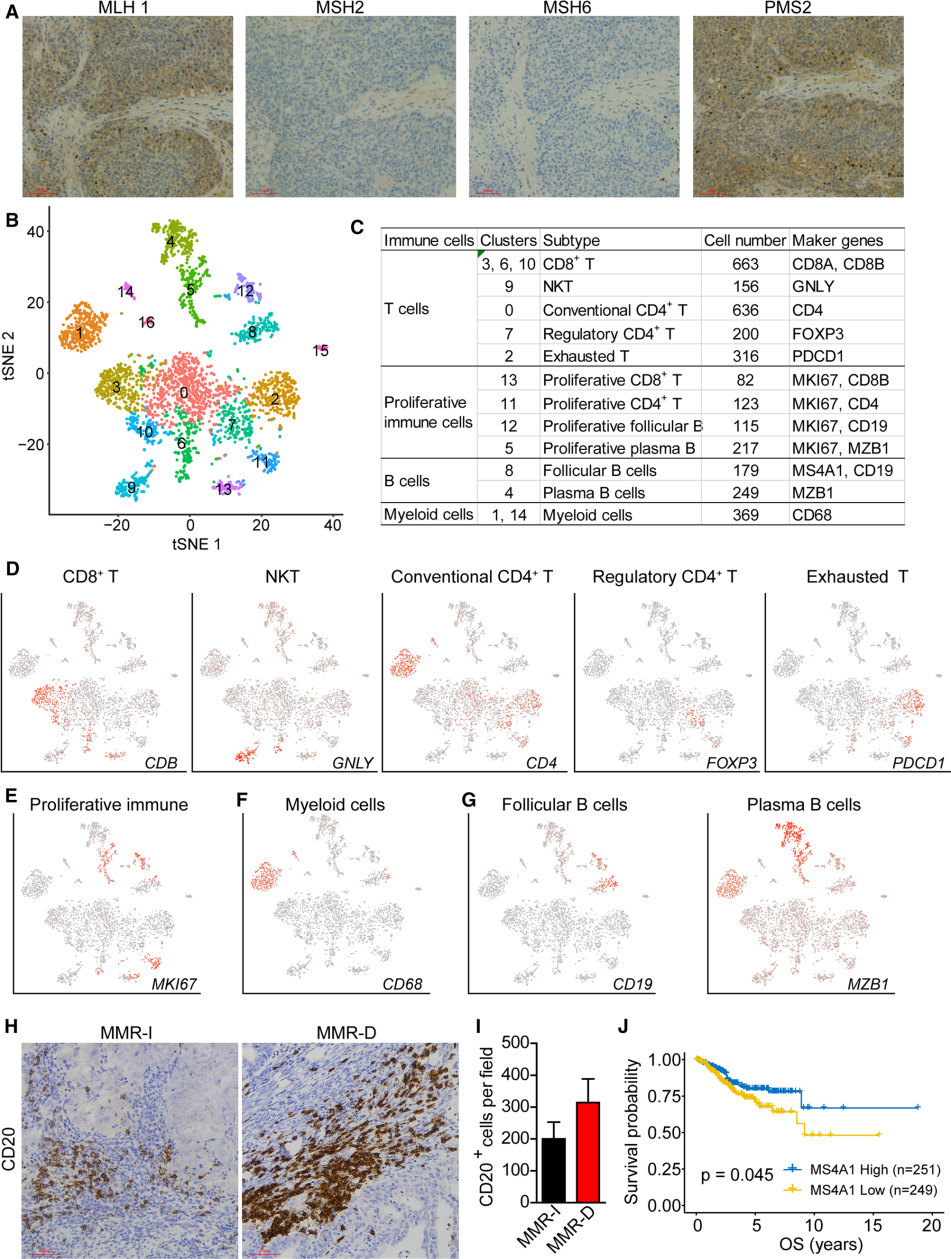
Atlas of multiple immune cell types in an MMR-D endometrial carcinoma sample (EC-MSI) and enrichment trend of B cells in MMR-D endometrial tumors. **A** IHC staining images of MLH1, MSH2, MSH6 and PMS2 in tumor slides isolated from the MMR-D sample. Scale bars, 60 μm. **B** t-SNE projection of all cells, color-coded by their associated cluster, and labeled with cluster number. **C** Cell cluster, number and marker genes of annotated immune cell types are summarized. **D**–**G** t-SNE map, colored by relative expression (lowest expression to highest expression, gray to red) of marker genes for immune cell subtypes: T cells (**D**), proliferative immune cells (**E**), myeloid cells (**F**), and B cells (**G**). **H** Representative IHC staining images of CD20 in slides isolated from MMR-D and MMR-I endometrial carcinoma sections. Scale bars, 60 μm. **I** Quantification of the numbers of B cells as presented in (**B**). Data are means ± SEM (15 MMR-D sections and 15 MMR-I sections were analyzed). Student’s t-test. **J** The overall survival curves with log-rank test for MS4A1 (CD20) expression based on TCGA-UCEC data. P value < 0.05 were considered statistically significant

Our previous study also indicated higher lymphocytes infiltration in an MMR-D cancer tissue compared to other (MMR-I) samples [20]. To gain deeper insight into the immune cellular composition and phenotypic diversity of MMR-D endometrial cancer, we profiled 3371 cells obtained from an MMR-D EC sample (labeled “EC-MSI”) using the single-cell RNA-seq (10X Genomics platform). The immunohistochemistry (IHC) staining of the mismatch repair (MMR) proteins showed that MSH2 and MSH6 expression were negative (Fig. 5A). The single-cell RNA–seq data was subjected to quality filtering and downstream analysis using Seurat R (version 2.3.4) package. Graph-based clustering (Fig. 5B) was used to classify cells into groups. We identified 4 major immune cell types through marker genes: proliferative immune cells, T cells, B cells, myeloid cells in this sample (Fig. 5C). The immune cells were further analyzed by identifying their subsets. T cells included conventional CD4^+^ T cells (cluster 0; *CD4*^+^), regulatory T cells (cluster 7; *FOXP3*^+^), CD8^+^ T cells (cluster 3, 6 and 10; *CD8B*^+^), exhausted T cells (cluster 2; *PDCD1*^+^) and natural killer T cells (NKT cells) (cluster 9; *GNLY*^+^) (Fig. 5C, D). Proliferative immune cells (*MKI67*^+^) consisted of both T and B cell lineages, including: proliferative CD4^+^ T cells (cluster 11), proliferative CD8^+^ T cells (cluster 13), proliferative follicular B cells (cluster 12) and proliferative plasma B cells (cluster 5) (Fig. 5C and E). Two clusters (cluster 1 and 14) of myeloid cells were characterized by enriched expression of CD68 (Fig. 5F). Two subtypes in B cells were further analyzed. Follicular B cells were (cluster 8) enriched for the expression of CD19 and MS4A1 [25], while plasma B cells (cluster 4) were characterized by the expression of MZB1 (Fig. 5C, G) [26]. We also reanalyzed the single-cell RNA–seq data of another MMR-D sample (EC5-T) published before [20] and decoded the immune ecosystem (Figure S2). We found that the two samples showed similar immune phenotypic diversity (Figure S2). These results formed the basis description of the immune subsets in endometrial carcinomas with MSI.

Brooke E et al. reported that the MSI tumors exhibited higher infiltration of CD3^+^ and CD8^+^ T cells compared to MSS tumors [27]. However, little is known about the relationship between MSI status and tumor infiltrating B cells. Here, we assessed the infiltration of B cells by IHC staining of MMR-D and MMR-I tumor sections with CD20 antibody (Fig. 5H). The MMR-D tumors exhibited a higher trend in the number of B cells than MMR-I tumors, but with no significance (Fig. 5I). Patient overall survival analysis from TCGA-UCEC data indicated that higher expression of CD20 (MS4A1) was associated with good prognosis (Fig. 5J). The function of B cells in endometrial carcinomas remains to be further explored.

## Discussion

Distinct factors affect the prognosis of endometrial cancer such as tumor type, grade and stage status [28, 29]. MMR-D subtype has been reported to be related with a good prognosis in endometrial cancer patients [14]. Moreover, promising clinical trials of immunotherapy (anti-PD-1/anti-PD-L1) indicate good response in advanced MMR-D/MSI-H EC patients [30, 31]. But the underlying mechanism between the MMR-D status and endometrial cancer patient prognosis is not well investigated and understood. In this study, we related the molecular subtype to clinical data and prognosis of EC patients based on 78 POLE, 123 MSI and 299 Other EC samples in the TCGA-UCEC project. The tumors in MSI subgroup were identified more often in early stage, had a lower age, better patient survival and enriched immune infiltrates than the Other subgroup. Besides, using the unbiased single-cell RNA-seq analysis for an MMR-D endometrial cancer tissue, an immune atlas was established.

Tumors with defects in MMR proteins indicated higher mutation frequency, and were suggested to harbor more tumor-specific neoantigens and higher lymphocytes infiltrates [24]. Here, our enrichment analysis of up-regulated genes in POLE and MSI subtype also suggested the enrichment of immune-related functions. Diversity of tumor infiltrating immune cells in tumor influences tumor development, progression, and treatment response to targeted agents. CD8^+^ T cells are essential for successful tumor killing. Regulatory T cells suppress anti-tumor immune response, and are usually associated with poor clinical outcomes [32]. A previous study reported that tumor-associated macrophages (TAMs) revealed a pro-tumor role in endometrial cancer [33]. Dendritic cells could be modulated by tumor cells, and thus drive immune tolerance [34]. In this study, both CD8^+^ T cells and regulatory T cells showed high infiltration in MSI subtype and were related with good OS. M2 macrophages and activated dendritic cells were found to be negatively correlated with MSI subtype and patient prognosis. The high infiltration of regulatory T cells might result from high neoantigens. Further investigations are necessary to clarify the exact functions of regulatory T cells and activated dendritic cells in EC.

B cells are the other major type of tumor-infiltrating lymphocytes (TILs) besides T cells. Several studies unveil that B cells suppress the progression of tumor. B cell depletion enhanced melanoma growth in mice [35]. Activation of B cells play an anti-tumor role by secreting immunoglobulins, promoting T cell response, and killing cancer cells directly [36, 37]. In this study, we tested MMR status of 98 EC patients, and identified 15 MMR-D cases. By comparing the infiltration of CD20^+^ B cells between 15 pairs of MMR-D and MMR-I EC tumors, we found that the MMR-D tumors showed a higher trend in the number of CD20^+^ B cells and that higher expression of CD20 (MS4A1) was associated with good prognosis. Two major subtypes of B cells and their versatile functions in non-small cell lung cancer have been reported [38]. Studies on B cell subtypes in EC are sparse, and the subsets and functions of B cells in the tumor microenvironment (TME) of EC remain largely unknown. We found two subtypes of B cells (follicular B cells and plasma B cells) in MMR-D EC, while the functions of different B cell subtypes necessitating further investigations to answer.

Tumors are complex ecosystems characterized by different compositions and functions of tumor-infiltrating immune cells and other stromal cells [39]. The TME characteristics plays a critical role in the responsiveness of immunotherapy [40]. Single-cell RNA-seq technology allowed for the in-depth profiling of heterogeneous immune cell populations. In this study, we provided a baseline description of the immune transcriptomes by single-cell RNA-seq analysis for an MMR-D EC tumor, which formed the basis for further examination into the role of immune subsets in endometrial carcinomas. Future studies are needed to determine the specific roles these complex immune cell types play in the regulation of EC development and progression.

## Supplementary Information

**Additional file1: Figure S1.** Subtype-related gene expression profile of 500 EC samples from TCGA database.** Figure S2.** IHC assessment of MMR status in an EC sample and marker annotation for major cell types in the tumor microenviroment.

**Additional file2: Table 1.** Upregulated genes in POLE and MSI groups compared with Other ECs.

## Data Availability

The single-cell sequencing raw data and processed data of EC-MSI have been deposited into the NCBI GEO database: GSE193430. Single-cell RNA-seq data of our published MMR-D sample (EC5-T) in PRJNA650549 were downloaded from the SRA database (https://www.ncbi.nlm.nih.gov/sra/PRJNA650549). All other data can be provided upon reasonable request to the corresponding author.

## Abbreviations

EC: Endometrial cancer
ECC: Endometrioid carcinoma
MMR: Mismatch repair
PTEN: Phosphate and tension homology deleted on chromosome ten
POLE: DNA polymerase ε
BMI: Body Mass Index

## Code availability

The code used in the current study are available from the corresponding author on reasonable request.
