# Supplementary material for: The clinicopathological characteristics, prognosis and immune microenvironment mapping in MSI-H/MMR-D endometrial carcinomas
Source: Discov Oncol. 2022 Mar 3;13:12. doi: 10.1007/s12672-022-00466-5 (PMC8894509; doi:10.1007/s12672-022-00466-5)
Supplement: Supplementary file 1 — Additional file1: Figure S1. Subtype-related gene expression profile of 500 EC samples from TCGA database. Figure S2. IHC assessment of MMR status in an EC sample and marker annotation for major cell types in the tumor microenviroment. [file 12672_2022_466_MOESM1_ESM.docx]

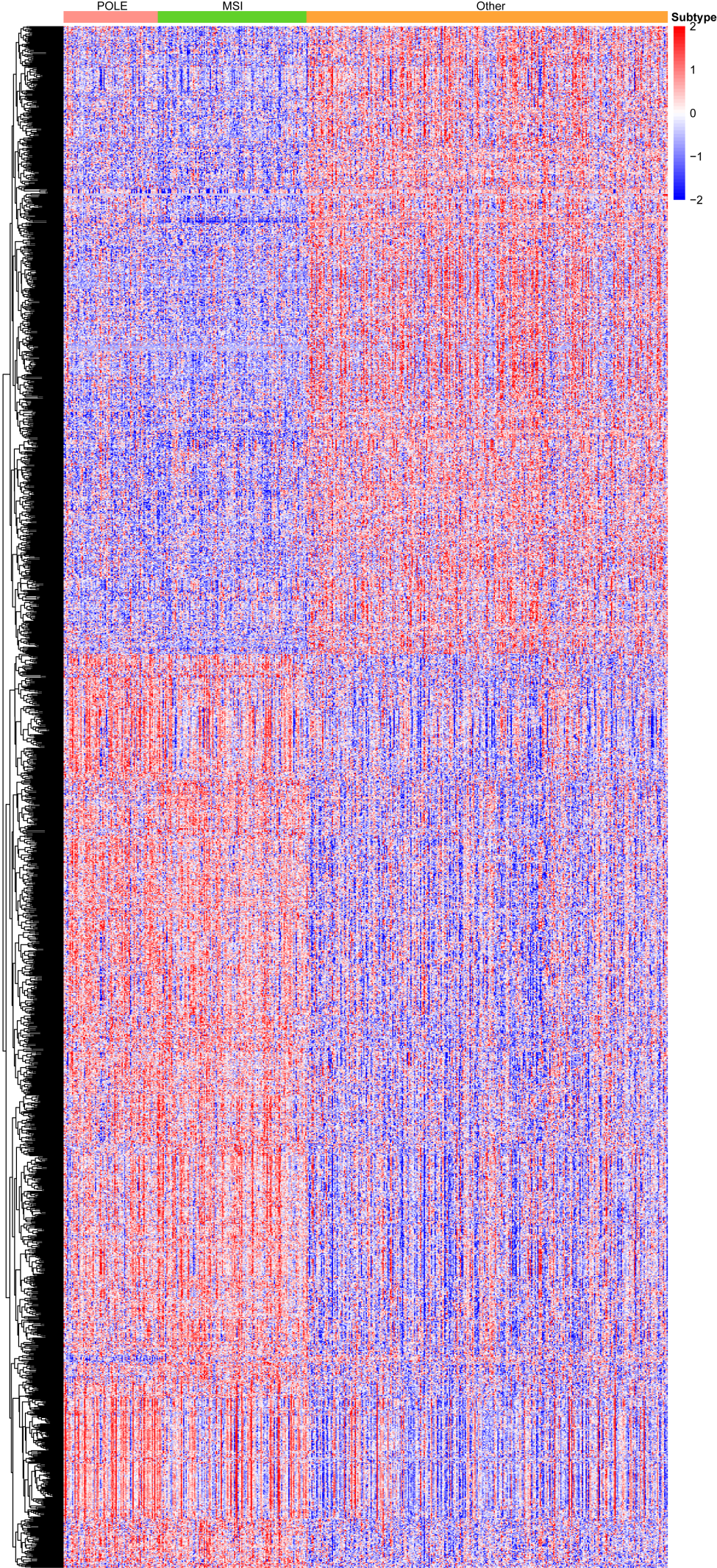


Figure S1. Subtype-related gene expression profile of 500 EC samples from TCGA database.


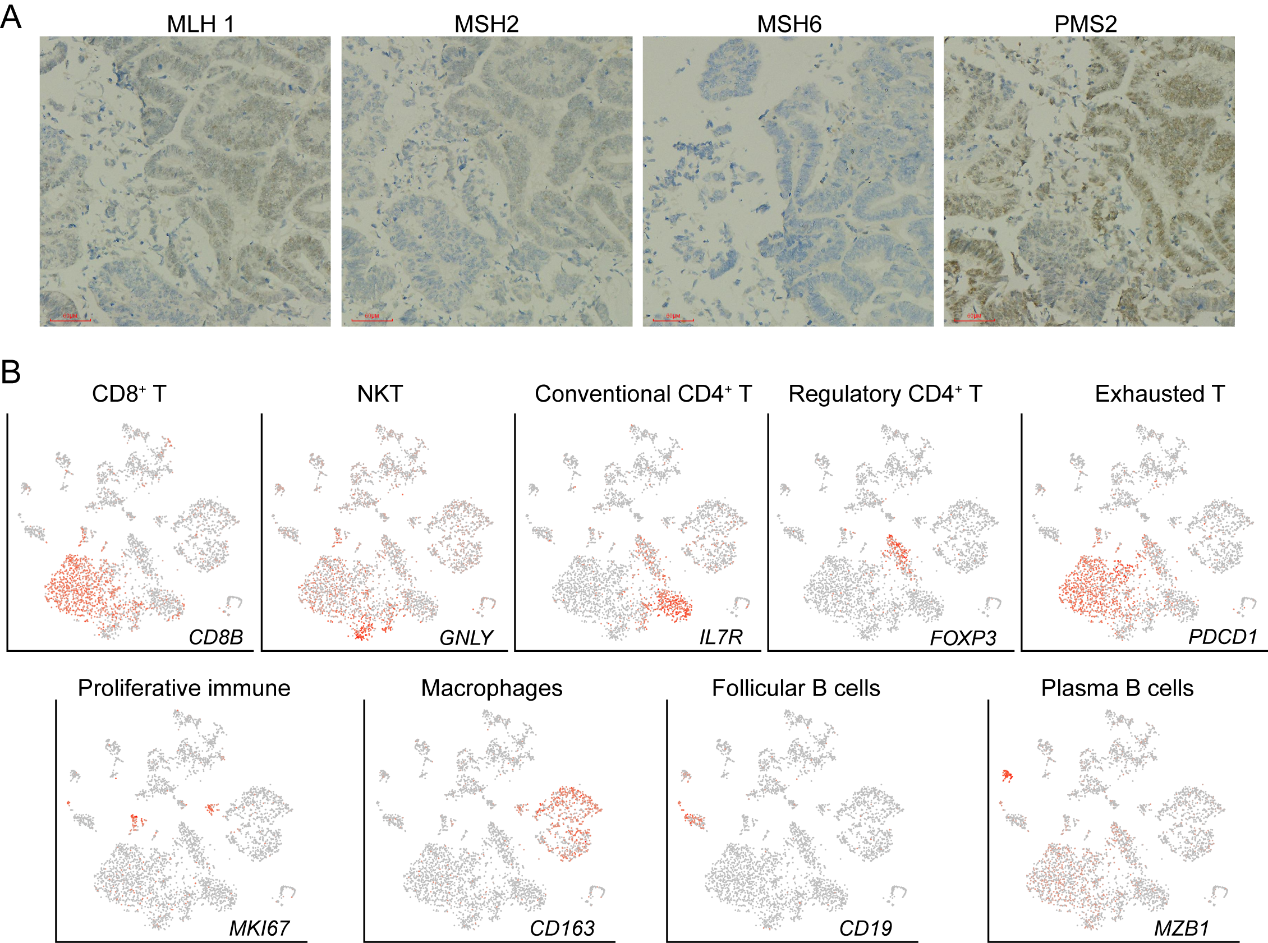


Figure S2. IHC assessment of MMR status in an EC sample and marker annotation for major cell types in the tumor microenviroment. (A) IHC staining images of MLH1, MSH2, MSH6 and PMS2 in tumor slides isolated from an MMR-D endometrial carcinoma section. Scale bars, 60 μm. (B) t-SNE maps showed expression levels (lowest expression to highest expression, gray to red) of representative markers for immune cell subtypes: T cells, proliferative immune cells, macrophages (F), and B cells.


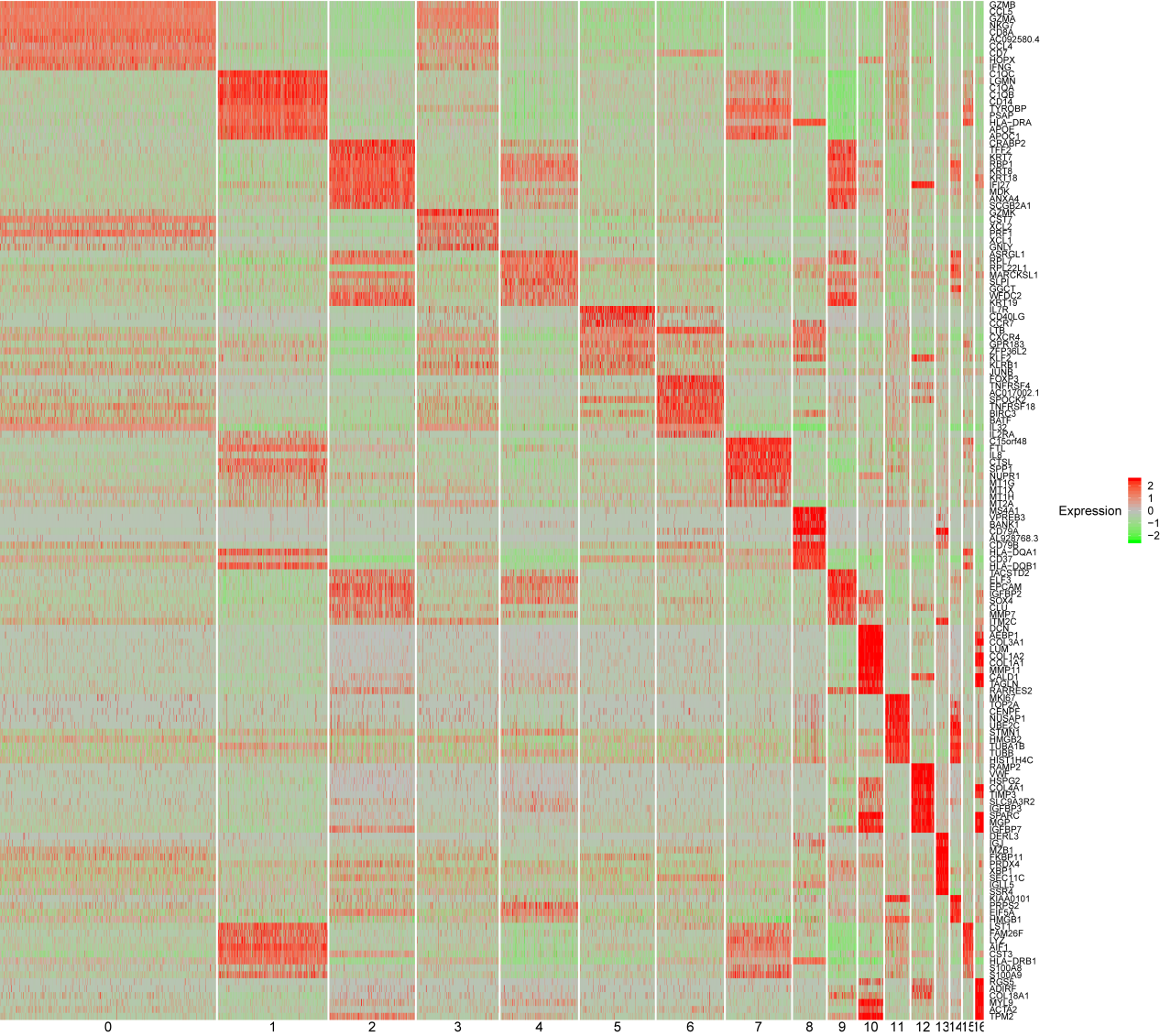


Figure S3. Heatmap showing expression levels of specific markers in each cell cluster.
